# Mediators of Racial Differences in COVID-19 Vaccine Acceptance and Uptake: A Cohort Study in Detroit, MI

**DOI:** 10.3390/vaccines10010036

**Published:** 2021-12-28

**Authors:** Abram L. Wagner, Lydia Wileden, Trina R. Shanks, Susan Door Goold, Jeffrey D. Morenoff, Sherri N. Sheinfeld Gorin

**Affiliations:** 1 Department of Epidemiology, School of Public Health, University of Michigan, 1415 Washington Heights, Ann Arbor, MI 48109, USA; 2 Department of Sociology, Gerald R. Ford School of Public Policy, Institute for Social Research, University of Michigan, Ann Arbor, MI 48109, USA; lwileden@umich.edu (L.W.); morenoff@umich.edu (J.D.M.); 3 School of Social Work, University of Michigan, Ann Arbor, MI 48109, USA; trwilli@umich.edu; 4 Department of Internal Medicine, Michigan Medicine, Ann Arbor, MI 48109, USA; sgoold@umich.edu; 5 Department of Family Medicine, Michigan Medicine, Ann Arbor, MI 48109, USA; ssgorin@med.umich.edu

**Keywords:** African Americans, vaccination coverage, minority groups, mediation analysis

## Abstract

Despite their disparate rates of infection and mortality, many communities of color report high levels of vaccine hesitancy. This paper describes racial differences in COVID-19 vaccine uptake in Detroit, and assesses, using a mediation model, how individuals’ personal experiences with COVID-19 and trust in authorities mediate racial disparities in vaccination acceptance. The Detroit Metro Area Communities Study (DMACS) is a panel survey of a representative sample of Detroit residents. There were 1012 respondents in the October 2020 wave, of which 856 (83%) were followed up in June 2021. We model the impact of race and ethnicity on vaccination uptake using multivariable logistic regression, and report mediation through direct experiences with COVID as well as trust in government and in healthcare providers. Within Detroit, only 58% of Non-Hispanic (NH) Black residents were vaccinated, compared to 82% of Non-Hispanic white Detroiters, 50% of Hispanic Detroiters, and 52% of other racial/ethnic groups. Trust in healthcare providers and experiences with friends and family dying from COVID-19 varied significantly by race/ethnicity. The mediation analysis reveals that 23% of the differences in vaccine uptake by race could be eliminated if NH Black Detroiters were to have levels of trust in healthcare providers similar to those among NH white Detroiters. Our analyses suggest that efforts to improve relationships among healthcare providers and NH Black communities in Detroit are critical to overcoming local COVID-19 vaccine hesitancy. Increased study of and intervention in these communities is critical to building trust and managing widespread health crises.

## 1. Introduction

More than 18 months after the first case of the coronavirus disease 2019 (COVID-19) was identified in the U.S., COVID-19 and its variants continue to spike cases and mortality rates across the country and around the globe. Research has shown that COVID-19 has disproportionately affected racial and ethnic minority groups [1]. Despite their disparate rates of infection and mortality, many communities of color report high levels of vaccine hesitancy.

Within the US, Wayne county, Michigan—home to Detroit—ranks 22nd in the number of confirmed COVID-19 cases and 8th in deaths, with a case to fatality ratio of 2.40% [2]. In Detroit, where more than 78% of the population is non-Hispanic (NH) Black and more than one third (36%) living in poverty [3], there have been nearly 52,000 confirmed COVID-19 cases and 2312 deaths. Detroit remains a COVID-19 case and mortality “hot spot”, yet vaccine uptake remains low. As of 28 July 2021, vaccination coverage within Detroit is 33%, compared to 54% for the state as a whole [4].

Low COVID vaccine uptake is not unique to Detroit. A number of international and national surveys have reported population-wide findings on the intention to take COVID-19 vaccines, which is a strong predictor of vaccination behavior [5]. A U.S. survey from March 2021 by Kaiser found that 62% of Americans have been vaccinated or intend to be, including 55% of non-Hispanic Black adults, 61% of Hispanic adults, and 64% of NH white adults [6].

Vaccine hesitancy is also not unique to COVID-19. The seasonal flu vaccine is the closest analogue to what an optional COVID-19 vaccine program could look like. The average adult uptake of the seasonal flu vaccine throughout the U.S., and within Michigan, during 2019–2020 was approximately 48% [7]. Coverage is about 8–9% lower in the NH Black population than NH whites [8]. Although past research has identified that disparities in vaccination behaviors and vaccine hesitancy exist by race and ethnicity [9], to our knowledge, few have rigorously studied the causal mechanisms behind vaccine hesitancy among those most affected—urban NH Black and Hispanic populations [10]. Moreover, the current COVID-19 pandemic offers a unique perspective on disparities faced within certain populations as a result of the scale and reach of this public health crisis.

To study minority communities’ receipt of a COVID-19 vaccine, we draw on a unique resource: a robust survey of Detroit fielded throughout the COVID-19 pandemic, including prior to the wide-scale implementation of vaccination programs. We describe racial/ethnic disparities in COVID-19 vaccine uptake in Detroit. We systematically assess, using a mediation model, if individuals’ personal experiences with COVID-19 and trust in health authorities prior to vaccine roll-out mediate racial and ethnic disparities in vaccination uptake. We hypothesize that experience with COVID-19 and trust in healthcare providers and/or government health officials are important mediators of racial and ethnic differences in vaccine uptake. Given continued sub-optimal uptake of the COVID-19 vaccine, these findings can continue to inform current efforts to reduce COVID-19 vaccine hesitancy.

## 2. Materials and Methods

### 2.1. Data

The Detroit Metro Area Communities Study (DMACS) is a panel survey of a representative sample of Detroit residents launched in 2016. The original panel of respondents was drawn from an address-based probability sample of all occupied Detroit households. DMACS was designed to descriptively estimate a number of different indicators in a precise fashion; the margin of sampling error for any indicator would be +/− 2.9 percentage points at the 95% confidence level, though this would vary by indicator due to the complex sample design. In subsequent years, the panel has been refreshed through additional address-based sampling. Starting in March 2020, less than 3 weeks after the CDC declared the COVID-19 pandemic a national emergency and with Detroit emerging as a “hot spot”, DMACS launched a series of rapid response surveys to understand Detroiters’ experiences with COVID-19. Due to restrictions on face-to-face interactions during the pandemic, outreach was limited to a subset of DMACS panelists who had previously provided email addresses and/or phone numbers as contact information. This paper focuses on data collected in the fifth rapid response COVID-19 survey, when a total of 1641 DMACS panelists were invited to participate. Eligible participants were adult residents of Detroit; 1012 surveys were completed, for a response rate of 62% (using AAPOR Response Rate 1). Surveys were self-administered online and interviewer-administered by telephone between 14 October and 28 October 2020. Between 2 June and 9 July 2021, individuals were followed up (N = 856, 83% of October wave) and asked about actual vaccination uptake. Data have been weighted to reflect the population of the city of Detroit. The questionnaires are available at: https://detroitsurvey.umich.edu/findings/ (accessed on 15 September 2021).

### 2.2. Vaccine Uptake as the Outcome Measure

Our main outcome is COVID-19 vaccine uptake, which we measured as initiation of at least one dose of any vaccine by the June 2021 wave. We also measured vaccine intent in October 2020 with the question “How likely are you to get a government-approved COVID-19 vaccine when it becomes available?” Responses were captured using a 4-point Likert scale that ranged from “very unlikely” to “very likely”. Following related, international studies of COVID-19 [11], we dichotomize this variable to capture if a respondent says they intend to vaccinate or not. Those who stated that they were somewhat or very likely to get vaccinated were categorized as intending to be vaccinated, while those who said somewhat or very unlikely were categorized as unlikely to be vaccinated.

### 2.3. Race/Ethnicity

Our primary independent variable of interest in this paper is respondents’ self-identified race/ethnicity, which we separated into Non-Hispanic Black, NH white, Hispanic, and NH other, in which we included all other categories, including Asian American, Pacific Islander, or Native American.

### 2.4. Mediators

We considered mediation in terms of six different measures of the participant’s personal experience with COVID-19, including two of trust: perceived severity of the COVID-19 pandemic, if the respondent had friends or family who had become ill with or who died from COVID-19, if the respondent had been diagnosed with COVID-19, the degree of the respondent’s trust in healthcare providers, and the degree of the respondent’s trust in government health officials.

### 2.5. Covariates

Other covariates in this model were selected based on their relevance to previous literature [11,12,13] and the Health Belief Model [14]. The Health Belief Model has posited that individuals will engage in a health behavior, for example, vaccination, if they receive cues to action. These cues may include a recommendation to vaccinate from a healthcare provider.

## 3. Statistical Analysis

Our analysis proceeds in four steps. First, we summarize the sociodemographic characteristics, COVID-19 experiences, trust in authorities, and vaccination intent, stratified across four racial/ethnicity groups.

We model the association between race and ethnicity on vaccination uptake using multivariable logistic regression. Coefficients are captured as odds ratios (ORs) and 95% confidence intervals (CI). To examine how vaccine intent by race/ethnicity varies with other sociodemographic characteristics, we include interactions between race/ethnicity and gender, race/ethnicity and education, and race/ethnicity and income.

To examine the relative importance of experiences with COVID-19 and trust in healthcare providers and officials on an individual’s intention to get vaccinated, we use Rao–Scott Chi-Square tests to test for significant differences across race/ethnicity.

Finally, using mediation models, we assess the influence of our independent variables on COVID-19 vaccine uptake in a series of multivariable logistic regression models. These models are limited to NH Black and NH white Detroiters in order to more precisely deconstruct mediating pathways. Each model separately examined the effect of our six measures of personal experience with COVID-19 and trust in healthcare providers and health authorities, adjusting for gender, age, income, and education. We did not put multiple measures of experiences and trust into the same model, as our a priori consideration was that there would be complex causal processes linking these together, and we wanted to estimate the total effect for each measure. For each of these models, we introduced an interaction term between race/ethnicity and every independent variable or covariate in order to examine differences in the association between independent variables and vaccine uptake by race. We present the results stratified by race/ethnicity.

Subsequently, we tested if differences in intent to vaccinate by race/ethnicity were mediated by experiences during COVID-19 or by trust in healthcare providers and health authorities, using the CAUSALMED procedure in SAS [15]. We report the proportion of the race/ethnicity–vaccination uptake relationship mediated and proportion eliminated by these mediating variables. Briefly, the proportion mediated comes from the natural pathway between the exposure (race/ethnicity) and the outcome, and represents what would happen to the strength of association between the exposure and outcome if we could disable the pathway through the mediator. The proportion eliminated is a policy-relevant measure, which represents what would happen to the strength of association between the exposure and outcome if we shifted everyone to counterfactually having the same value for the mediator [16]. All analyses were conducted in SAS version 9.4 (SAS Institute, Cary, NC, USA), with listwise deletion of missing data, and with an alpha level of 0.05. Our reporting of the study’s findings follows the STROBE guidelines for cross-sectional studies (see Supplementary Table S1).

## 4. Ethical Review

The protocol was approved by the University of Michigan Institutional Review Board (#HUM00112364). Participants read over an informed consent form and agreed to it electronically prior to any data collection.

## 5. Results

Of the 1012 respondents in the October 2020 wave, the majority, 77% (N = 714), identified as NH Black, and the rest identified as NH white (10%), Hispanic (8%), or other (5%) (Table 1). Within Detroit, 39% said they intended to get vaccinated once the vaccine became available. NH Black Detroiters expressed the lowest intention to be vaccinated: only 32% of NH Black residents reported being willing to vaccinate in October 2020, compared to 69% of NH white Detroiters, 51% of Hispanic Detroiters, and 45% of other racial/ethnic groups. A total of 856 (83%) were able to be followed up in the June 2021, with no significant difference in loss-to-follow-up by race/ethnicity. As of June 2021, 58% had received at least one dose of a COVID-19 vaccine, with uptake highest in NH white Detroiters (82%).

NH Black individuals perceived greater severity of COVID-19 than other subgroups, although Hispanics reported more friends and family having become ill from COVID-19 than other groups. Across the racial/ethnic subgroups, few had been diagnosed with COVID-19. NH Black Detroiters reported the largest number of deaths from COVID-19 among family and friends (41% reported this experience, vs. 7% of NH white Detroiters, *p* < 0.0001). Trust in healthcare providers significantly differed across racial/ethnic groups (*p* = 0.0013) and was greatest among NH whites; trust in government was similar across the racial/ethnic groups included in this study (*p* = 0.4161).

Table 2 examines the relationship between race and vaccination uptake, and the interaction between race and other sociodemographic variables on vaccination. Vaccination was relatively low in the NH Black population, and higher in other race/ethnic groups (e.g., in NH white vs. NH Black: OR = 3.24, 95% CI: 3.14, 3.33). We also found that vaccine uptake varied significantly by gender, education, and age.

Among NH Black Detroiters, the odds of vaccination were higher in females than males (OR: 1.03, 95% CI: 1.01, 1.04), in those with a college education compared to less schooling (OR: 2.00, 95% CI: 1.94, 2.06), and in those with an income ≥USD 50,000 vs. less (OR: 1.98, 95% CI: 1.94, 2.02). The interactions between race and gender, education, or income were all statistically significant. In the aggregate, findings from this interaction analysis point to young, male, NH Black Detroiters with lower education levels and lower income as being less likely to obtain a vaccine, whereas other racial/ethnic groups had different patterns.

Table 3 explores differences in the major predictors of vaccination intention by sub-groups of residents. The only factor that produced a statistically significant difference in vaccine intent across racial groups was a recommendation from a healthcare provider (*p* < 0.0056). Most (90%) NH white Detroiters were influenced in their intention to vaccinate by their healthcare providers, compared to 74% of Hispanic Detroiters, 77% of others, and 67% of NH Black Detroiters. Differences in personal experience with COVID-19 did not produce significant differences in the likelihood of vaccinating.

In six multivariable regression models with the six measures of COVID-19 personal experiences and perceived trust in healthcare providers and health authorities (Table 4), we found that, overall, most of these measures were significantly related to vaccine uptake, but the strength of association differed between NH Black and NH white Detroiters. For example, in white Detroiters, perceived severity of COVID-19 was significantly associated with vaccine uptake (OR: 2.30, 95% CI: 2.21, 2.39), but this association was null for Black Detroiters (OR: 0.98, 95% CI: 0.94, 1.01). Similarly, having friends and family who had died from COVID-19 was a pre-disposing factor for NH white Detroiters but the opposite was so for NH Black Detroiters. For trust in healthcare providers and trust in government health officials, there were stronger, positive associations for Black Detroiters than among other racial/ethnic subgroups.

Trust in healthcare providers explained 6% of the differences in vaccine uptake between NH Black and NH white Detroiters. The mediation analysis revealed that 23% of the differences in vaccine uptake by race could be eliminated if NH Black Detroiters were to have levels in trust in healthcare providers similar to those among white NH Detroiters. Trust in government health officials showed a similar pattern: there was a minimal (3%) amount of mediation. Importantly, increasing the levels of trust in NH Black Detroiters to those observed in white Detroiters could eliminate 18% of the difference in vaccine uptake.

## 6. Discussion

Using a robust, probability-based sample from Detroit, we evaluated the mediators of COVID-19 vaccine to better understand the causes of vaccine hesitancy in a multi-ethnic, multi-racial urban sample with high vaccine hesitancy. We are among the first to systematically assess, using causal models, the impact of these mediators, including the personal, lived experience, on separate ethnic and racial subsamples of an urban population. We found less influence of perceived COVID-19 severity on vaccination among NH Black Detroiters by comparison to their NH white counterparts. Trust in a healthcare provider and trust in government health officials were stronger predictors of vaccine uptake among NH Blacks relative to NH whites; however, levels of trust in healthcare providers were relatively low among NH Black Detroiters. Through the mediation analysis, we identified a substantial impact of trust on mediating the difference in uptake between NH Blacks and NH whites. These findings could undergird policy approaches, public health communications, and community- and individual-level intervention approaches that are targeted to specific population subgroups to decrease vaccination hesitancy.

The published literature on COVID-19 vaccine uptake has found that higher hesitancy is associated with younger age [12,13,17]. We also found that higher income NH Blacks were more likely to intend to vaccinate relative to NH Blacks with lower income. Greater resources generally confer increased access to healthcare; these advantages do not necessarily accrue equally to Blacks and whites, particularly in communities with a history of structural racial inequities such as Detroit. Our interaction analysis reveals more income-based disparities among NH Black Detroiters than other races or ethnicities. Further, in the early phase of the pandemic, there were inconsistent public health messages about the importance of vaccines, the likelihood of Operation Warp Speed producing enough vaccines, the mechanisms for obtaining a vaccination, and the number and type of vaccination sites, which may have reduced the impact of wealth on COVID-19 vaccination intent among NH Black respondents. Perceptions about vaccine effectiveness are also important for determining willingness to be vaccinated [18]; the speed of vaccine development could influence how effective or safe individuals think these vaccines are.

Trust in healthcare providers and governmental health officials emerged as an important reason behind vaccine acceptance among those groups most adversely affected by COVID-19. Black Detroiters are at increased risk of being infected, as well as suffering from negative direct or indirect consequences of COVID-19 [19,20]. There is an urgent need for policy and community-based approaches that could be measured by the benchmarks we specified in this study, for example, to decrease vaccine hesitancy among NH Black Detroit residents. Additional public health research could explore the determinants of hesitancy over time, using culturally sensitive measures, as there could also be many alternative reasons why NH Black and NH white Detroiters may have differences in vaccine uptake, including issues of access. These could include difficulties in transportation, time costs for obtaining a vaccine, and lack of insurance [21,22,23]. Addressing these inequities will require investments in areas such as increasing vaccine subsidies and insurance coverage, more local vaccine sites, as well as testing culturally and linguistically sensitive vaccination messages.

We note that reasons for COVID-19 vaccine uptake and hesitancy can vary widely and the results from our study in Detroit do not necessarily translate to other populations. However, research from across the world has found that hesitancy is greater in lower income countries [24] and poorer communities within countries [25]. COVID-19 vaccine acceptance and uptake could also be affected by social media, mistrust of the government in general, and attitudes towards the pharmaceutical industry [26]. Future research could also provide more detail into the reasons why individuals are hesitant, and what reasons are most important.

## 7. Limitations

A strength of our study was the robust probability-based study that offered us the ability to look at a large population of NH Black Americans and compare their responses to other subgroups who are living in the same urban settings. We also measured our mediating variables prior to the outcome, vaccine uptake. However, we examine a limited set of potential mediators or reasons behind vaccine hesitancy. We also examined the COVID-19 personal experiences and trust as separate mediators, but they could also be examined in future research at the same time in a larger model. Additionally, there could be biases in loss to follow up, which could affect the generalizability of the results. Prior influenza vaccination is a strong predictor of adult COVID-19 vaccination; we did not measure this factor in the survey so cannot assess its influence in this sample. There also could be other unmeasured confounders. We note that our measure of uptake was slightly higher than contemporary measures from the Michigan Department of Health and Human Services, although this could be due to bias from our survey or from the state records [27].

## 8. Conclusions

Given the estimated required population vaccination threshold of 70–80% for herd immunity, our analyses demonstrated the need for increased study of, and intervention in, the NH Black Detroit community. Our analyses suggest the critical importance of trust—in healthcare providers and government health communications and leaders—for vaccine acceptance among those subgroups most adversely affected by COVID-19. Future research could provide more context into the reasons behind vaccine hesitancy and their relative strength. Yet, findings from this study reveal that improved community relationships between healthcare providers and Black residents could reduce COVID-19 vaccine hesitancy. With the pandemic threat still active, the need is urgent.

## Figures and Tables

**Table 1:** Demographic characteristics, COVID-19 experiences, trust in healthcare providers and authorities, and vaccination intent, stratified by race/ethnicity, Detroit Metro Area Community Study, October 2020 and June 2021.

		Overall	In NH Black Detroiters	In NH White Detroiters	In Hispanic Detroiters	In Other Detroiters	*p*-Value
Overall (row %)			714 (77%)	129 (10%)	57 (8%)	112 (5%)	
Gender	Male	287 (47%)	167 (43%)	55 (71%)	12 (46%)	53 (61%)	<0.0001
	Female	723 (53%)	547 (57%)	74 (29%)	45 (54%)	57 (39%)	
Age	18–24	61 (11%)	31 (9%)	5 (5%)	14 (38%)	11 (17%)	<0.0001
	25–34	178 (23%)	100 (19%)	38 (43%)	18 (35%)	22 (19%)	
	35–44	183 (17%)	114 (16%)	34 (24%)	13 (16%)	22 (18%)	
	45–54	189 (14%)	154 (16%)	7 (5%)	8 (8%)	20 (14%)	
	55–64	223 (15%)	178 (17%)	22 (11%)	4 (4%)	19 (15%)	
	≥65	168 (19%)	130 (23%)	23 (12%)	0 (0%)	15 (16%)	
Yearly income	<USD 50,000	655 (69%)	482 (75%)	62 (36%)	38 (61%)	73 (74%)	<0.0001
	≥USD 50,000	276 (31%)	178 (25%)	59 (64%)	17 (39%)	22 (26%)	
Education	<Bachelor’s degree	702 (83%)	522 (88%)	52 (45%)	50 (89%)	78 (81%)	<0.0001
	≥Bachelor’s degree	306 (17%)	192 (12%)	76 (55%)	7 (11%)	31 (19%)	
Perceived severity of COVID-19	Very serious	695 (69%)	537 (75%)	58 (39%)	36 (56%)	64 (62%)	<0.0001
	Somewhat serious	235 (21%)	139 (19%)	51 (36%)	12 (24%)	33 (22%)	
	Not too serious	61 (7%)	23 (3%)	18 (22%)	7 (18%)	13 (15%)	
	Not at all serious	13 (2%)	9 (2%)	1 (2%)	2 (2%)	1 (1%)	
	Don’t know	4 (1%)	3 (1%)	1 (0%)	0 (0%)	0 (0%)	
Friends/family ever ill from COVID-19	No	457 (47%)	317 (47%)	63 (57%)	22 (37%)	55 (45%)	0.2464
	Yes	555 (53%)	397 (53%)	66 (43%)	35 (63%)	57 (55%)	
Friends/family died from COVID-19	No	626 (64%)	399 (59%)	114 (93%)	38 (72%)	75 (66%)	<0.0001
	Yes	386 (36%)	315 (41%)	15 (7%)	19 (28%)	37 (34%)	
Ever diagnosed with COVID-19	No	971 (96%)	680 (96%)	126 (98%)	55 (94%)	110 (99%)	0.4125
	Yes	41 (4%)	34 (4%)	3 (2%)	2 (6%)	2 (1%)	
Trust healthcare provider about COVID-19	Not at all	62 (7%)	46 (7%)	1 (0%)	6 (11%)	9 (6%)	0.0013
	A little	355 (36%)	250 (39%)	37 (27%)	18 (28%)	50 (48%)	
	A great deal	581 (56%)	407 (54%)	90 (73%)	33 (61%)	51 (46%)	
Trust government about COVID-19	Not at all	158 (18%)	118 (19%)	17 (15%)	6 (13%)	17 (12%)	0.4161
	A little	420 (40%)	279 (38%)	50 (44%)	27 (48%)	64 (58%)	
	A great deal	423 (41%)	308 (42%)	62 (41%)	24 (39%)	29 (31%)	
Intent to be vaccinated (October 2020)	Very unlikely	416 (38%)	343 (44%)	15 (11%)	19 (25%)	39 (41%)	<0.0001
	Somewhat unlikely	218 (23%)	159 (25%)	25 (20%)	12 (23%)	22 (14%)	
	Somewhat likely	231 (24%)	144 (23%)	34 (22%)	22 (43%)	31 (28%)	
	Very likely	139 (14%)	61 (9%)	55 (47%)	4 (8%)	19 (17%)	
Included in June 2021 wave	Included	856 (83%)	611 (83%)	104 (81%)	51 (88%)	90 (81%)	0.7222
	Lost to follow up	156 (17%)	103 (17%)	25 (19%)	6 (12%)	22 (19%)	
At least one dose of COVID-19 vaccine (June 2021)	Yes	522 (58%)	356 (56%)	88 (82%)	27 (50%)	51 (52%)	0.0042
	No	325 (42%)	246 (44%)	16 (18%)	24 (50%)	39 (48%)	

Note: NH, non-Hispanic. Missing data are not included.

**Table 2:** Interactions between race/ethnicity and other sociodemographic variables in vaccine uptake among 784 Detroiters, June 2021.

Category	OR (95% CI)	*p*-Value	OR (95% CI)	*p*-Value
Race/ethnicity		<0.0001		<0.0001
NH Black	ref.		ref.	
NH white	3.24 (3.14, 3.33)		5.55 (5.26, 5.87)	
Hispanic	1.54 (1.50, 1.58)		2.74 (2.62, 2.86)	
Other	0.71 (0.69, 0.74)		0.70 (0.66, 0.74)	
Female vs. male	0.90 (0.89, 0.92)	<0.0001	1.03 (1.01, 1.04)	0.0011
Race × Gender Interaction				<0.0001
NH white × Female			0.32 (0.30, 0.34)	
Hispanic × Female			0.47 (0.45, 0.49)	
Other × Female			1.00 (0.93, 1.08)	
College vs. less education	2.61 (2.55, 2.67)	<0.0001	2.00 (1.94, 2.06)	<0.0001
Race × Education Interaction				<0.0001
NH white × College			3.80 (3.53, 4.09)	
Hispanic × College			1.23 (1.14, 1.34)	
Other × College			3.84 (3.44, 4.29)	
Income ≥ USD 50,000 vs. less	1.74 (1.71, 1.77)	<0.0001	1.98 (1.94, 2.02)	<0.0001
Race × Income Interaction				<0.0001
NH white × ≥USD 50,000			0.30 (0.28, 0.32)	
Hispanic × ≥USD 50,000			0.62 (0.59, 0.65)	
Other × ≥USD 50,000			0.56 (0.52, 0.62)	
Age		<0.0001		<0.0001
18–39 years	0.33 (0.33, 0.34)		0.32 (0.31, 0.32)	
40–64 years	ref.		ref.	
≥65 years	2.66 (2.60, 2.71)		2.61 (2.56, 2.67)	

Note: NH, non-Hispanic.

**Table 3:** Importance of additional factors in an individual’s intention to become vaccinated, stratified by race/ethnicity, and by gender among Black Detroiters, October 2021.

	Among All Detroiters				
	NH Black (%)	NH White (%)	Hispanic (%)	Other (%)	*p* ^a^
	714	159	57	112	
Country where vaccine produced	437 (61%)	76 (62%)	40 (74%)	68 (57%)	1
Recommendation from healthcare provider	473 (67%)	111 (90%)	40 (74%)	77 (69%)	0.0056
Recommendation from government health officials	371 (56%)	90 (68%)	38 (69%)	51 (50%)	0.7960
Vaccine used for long time with no serious side effects	568 (81%)	111 (82%)	47 (86%)	87 (83%)	1
COVID-19 risk of infection when vaccine is available	531 (75%)	93 (72%)	41 (70%)	78 (70%)	1
Time and place of vaccination	444 (66%)	70 (56%)	39 (67%)	65 (60%)	1
Vaccine is free	415 (62%)	67 (45%)	41 (65%)	67 (56%)	0.6376
Know other people getting vaccinated	351 (51%)	50 (39%)	34 (62%)	55 (53%)	1

Notes: NH, non-Hispanic. ^a^ *p*-value from Rao–Scott Chi-Square Test, controlled for multiple testing through a Bonferroni correction factor of 8.

**Table 4:** Odds of vaccine uptake by COVID-19 experiences and trust in healthcare providers and authorities, in NH Black and NH white Detroiters in the Detroit Metro Area Community Study, October 2020 and June 2021.

		Odds Ratio of COVID-19 Vaccine Uptake (95% CI)			Mediation of Race-Vaccination Uptake Relationship	
	N	In NH Black Detroiters	In NH white Detroiters	*p*-Value ^a^	% Mediated	% Eliminated
Perceived COVID-19 severe vs. not	654	0.98 (0.94, 1.01)	2.30 (2.21, 2.39)	<0.0001	−1%	54%
Friends/family ever ill from COVID-19 vs. not	656	1.45 (1.43, 1.48)	1.23 (1.19, 1.27)	<0.0001	0%	13%
Friends/family died from COVID-19 vs. not	656	0.76 (0.75, 0.77)	1.42 (1.36, 1.47)	<0.0001	2%	−2%
Ever diagnosed with COVID-19 vs. not	656	1.03 (0.99, 1.07)	0.04 (0.04, 0.05)	<0.0001	0%	0%
Trust healthcare provider a great deal vs. not	649	2.65 (2.61, 2.70)	1.45 (1.40, 1.49)	<0.0001	6%	23%
Trust government health officials a great deal vs. not	650	2.84 (2.79, 2.89)	0.74 (0.72, 0.77)	<0.0001	3%	18%

Notes: Each row represents a separate model; each model controlled for gender, age, income, and education as covariates. Mediators assessed in October 2020, outcome in June 2021. ^a^ for interaction of race and main effect, i.e., difference in strength of association between Black and white Detroiters. NH, non-Hispanic.

## Data Availability

The data are available through: https://doi.org/10.3886/ICPSR37687.v1 (accessed on 15 September 2021).

## Supplementary Materials

The following are available online at https://www.mdpi.com/article/10.3390/vaccines10010036/s1, Table S1: STROBE Statement—Checklist of items that should be included in reports of cohort studies.
